# Characterization of a Bioresorbable Magnesium-Reinforced PLA-Integrated GTR/GBR Membrane as Dental Applications

**DOI:** 10.1155/2020/6743195

**Published:** 2020-09-19

**Authors:** Xin Du, Yahui Song, Xinxin Xuan, Shuzhen Chen, Xia Wu, Heng Bo Jiang, Eui-Seok Lee, Xiaohui Wang

**Affiliations:** ^1^Stomatological Materials Laboratory, School of Stomatology, Shandong First Medical University & Shandong Academy of Medical Sciences, Tai'an, Shandong 271016, China; ^2^Jinan Stomatological Hospital, Jinan, Shandong 250001, China; ^3^Department of Oral and Maxillofacial Surgery, Graduate School of Clinical Dentistry, Korea University Guro Hospital, Seoul 08308, Republic of Korea

## Abstract

Inferior mechanical properties have always been a limitation of the bioresorbable membranes in GBR/GTR. This study is aimed at fabricating a bioresorbable magnesium-reinforced polylactic acid- (PLA-) integrated membrane and investigating its mechanical properties, degradation rate, and biocompatibility. The uncoated and fluoride-coated magnesium alloys, AZ91, were made into strips. Then, magnesium-reinforced PLA-integrated membrane was made through integration. PLA strips were used in the control group instead of magnesium strips. Specimens were cut into rectangular shape and immersed in Hank's Balanced Salt Solution (HBSS) at 37°C for 4, 8, and 12 d. The weight loss of the AZ91 strips was measured. Three-point bending tests were conducted before and after the immersion to determine the maximum load on specimens. Potentiodynamic polarization (PDP), electrochemical impedance spectroscopy (EIS), scanning electron microscopy (SEM), and energy-dispersive spectroscopy (EDS) were conducted on coated and uncoated AZ91 plates to examine corrosion resistance. Murine fibroblast and osteoblast cells were cultured on circular specimens and titanium disks for 1, 3, and 5 d. Thereafter, WST test was performed to examine cell proliferation. As a result, the coated and uncoated groups showed higher maximum loads than the control group at all time points. The weight loss of AZ91 strips used in the coated group was lower than that in the uncoated group. PDP, EIS, SEM, and EDS showed that the coated AZ91 had a better corrosion resistance than the uncoated AZ91. The cell proliferation test showed that the addition of AZ91 did not have an adverse effect on osteoblast cells. Conclusively, the magnesium-reinforced PLA-integrated membrane has excellent load capacity, corrosion resistance, cell affinity, and proper degradation rate. Moreover, it has great potential as a bioresorbable membrane in the GBR/GTR application.

## 1. Introduction

Periodontitis is a chronic inflammation of periodontal attachment tissues caused by local factors, which causes a destructive periodontal disease and alveolar bone loss [1, 2]. The regeneration of the periodontal tissue defect is a challenge for clinicians [3]. GBR/GTR is a radical treatment technique for the periodontal disease. The principle of the periodontal regeneration surgery is to use a membrane as the barrier material. The resistance of the surface to contact with the gingival connective tissue occupies a particular space. It guides the periodontal ligament cells to occupy the root surface of the tooth to form a new cementum and attachment [4].

Barrier membranes are fundamental for GTR/GBR. The ISO selects an ideal biomaterial for GTR/GBR membranes, which should fulfill the main design criteria, such as biocompatibility, space-making, cell occlusiveness, tissue integration, and clinical operability [2, 4]. These materials can be classified into nonbioresorbable and bioresorbable membranes, according to their degradation characteristics [4]. Bioresorbable membranes have the apparent advantage of degradation. However, its strength is too low to be used for alveolar bone absorption in a large area of posterior teeth, such as polylactic (PLA), collagen membrane [5], and chitosan [6]. Collagen is currently the most popular GTR/GBR membrane material that promotes bone regeneration [7]. However, two disadvantages of collagen membrane limit its development. On the one hand, collagen membrane has low mechanical strength and fast degradation rate *in vivo*. Hence, it easily collapses and loses space support in the application process. Postlethwaite et al. [8] discussed that collagen can act as a barrier to promote cell/tissue regeneration, with an average retention time of about 6 weeks and a relatively short supporting time. On the other hand, the raw material of collagen membrane comes from animal collagen, and the extraction purity is low. Therefore, production cost is expensive. In addition, because the collagen of animals may contain infectious pathogens carried from the animals, the collagen membrane can be infectious to some extent. Chitosan, also called acetyl chitosan, is derived from crustaceans, such as crabs, shrimps, and crayfish. It is a natural polymer material that is bioresorbable [9]. However, owing to the poor mechanical strength of chitosan membrane, it can only be used to treat alveolar bone resorption and periodontal problems in small areas. Moreover, there are many methods for making chitosan membranes, most of which are in the primary stage; therefore, further research is required. Rakhmatia et al. believed that the absorption degree of bioresorbable materials was unpredictable and could affect bone regeneration. If the bioresorbable membrane material was absorbed too quickly, it would lead to the formation of an incomplete barrier membrane structure. It means that the strength of the membrane material for surgeries was not enough to support the regeneration of periodontal tissues and bones [4, 10]. Chen et al. believed that the early exposure of nonbioresorbable GTR membranes to the mouth could lead to bacterial infection, leading to a failure or the incomplete regeneration of periodontal tissues or bones [6, 11]. Nonbioresorbable membranes have prominent strength; however, they cannot be absorbed in the body because they can easily cause secondary infection, such as titanium mesh and polytetrafluoroethylene (PTFE) [4, 6, 12]. In 1984, PTFE membranes were firstly introduced into the field of oral cavity [13]. According to the structure, PTFE is divided into expanded PTFE (e-PTFE) and high-density PTFE (d-PTFE). E-PTFE has ideal mechanical properties and can better maintain the growth space of periodontal tissues. E-PTFE membranes and bioresorbable membranes are usually required to be closed with sutures during the primary surgery to prevent the growth of soft tissues, bacterial infection, membranes location migration, and exposure of implants to the mouth. Nonetheless, d-PTFE membranes have a small aperture and high density, which can prevent various bacteria from infiltrating the barrier membranes and reduce the chance of infection.

Toygar et al. [14] reported that there was no significant difference in the adhesion of dental plaque between the titanium membrane and e-PTFE membrane in the treatment of periodontal defects. The results showed that the titanium membrane was equivalent to e-PTFE membrane for periodontal tissue regeneration and can be used for the treatment of periodontal defects. Although the titanium mesh is excessively hard, it can increase the exposure probability of the barrier membrane and increase the stimulation to these soft tissues [15]. The barrier membrane of a single material has different defects, but a composite barrier membrane made of various materials can achieve a mutually reinforcing effect, such as Ti-PTFE [3, 12]. With the help of Ti, the strength of the barrier membrane can be enhanced; however, Ti-PTFE is not a bioresorbable membrane and requires a second operation to be removed. Therefore, it is particularly essential to seek a composite barrier membrane with high strength and bioabsorbability in the current field of GTR/GBR.

Polylactic (PLA) is an aliphatic polyester with better biological activity, deriving from fully renewable resources such as corn and sugar beets [16, 17]. It has a relatively complete manufacturing process that uses a two-step method. First, lactide is obtained by the condensation reaction of lactic acid and then prepared by ring-opening polymerization of lactide [16]. A wide range of experimental studies have found that PLA materials have superior bioabsorbability [18]. After use, PLA can be completely degraded naturally by microorganisms and eventually generate carbon dioxide and water without polluting the environment, which is relatively favorable to environmental protection. As a surgical implant material, its degradability *in vivo* and *in vitro* has also been recognized [19]. This further confirms that PLA has excellent biocompatibility, excellent processability, and controllable permeability (for the access of nutrients into the bone defect). *In vitro*, it performs well in the adhesion of epithelial connective tissues and alveolar bone regeneration, with satisfactory periodontal tissue regeneration [20]. However, considering the inferior mechanical properties of polymeric materials, the individual application of polymers in several clinical situations, especially large bone defects, is limited. Nowadays, PLA has been widely applied in GTR/GBR surgery, which still has an objective prospect in medical biology.

Magnesium alloys as biodegradable implants have distinctive preponderances over Fe-based alloys and Zn-based alloys [21]. In the human body, the degradation product of magnesium alloys is Mg^2+^. Mg^2+^ is the coenzyme factor of many metabolisms in the body, and Mg^2+^ significantly promotes the formation of new bones [22]. Magnesium alloys produce hydrogen gas during the metabolic process, leading to a high rate of magnesium degradation, which increases the concentrations of Mg^2+^ in body fluids. However, a significant increase in the concentration of Mg^2+^ will inhibit the deposition of osteoblast mineral matrix, which in turn will reduce the osteoblast activity, thus resulting in osteomalacia-like performance [23]. The most effective way to slow down the corrosion rate of magnesium alloys is to perform surface modification, such as hydroxyapatite coating [24] and hydrofluoric acid coating [25–27]. Owing to the controlled permeability of PLA and the excellent mechanical properties of magnesium alloy AZ91, bonding the two together can complement each other. The magnesium alloy serves as the reinforcing core to support the PLA membrane and increase its strength. PLA, as a barrier, on the one hand, can prevent direct contact between magnesium alloy and the surrounding bone tissue, and on the other hand, it can reduce the leakage of Mg^2+^.

Therefore, the preparation of a magnesium alloy (AZ91)-reinforced PLA-integrated membrane has both degradability and functional strength. Furthermore, the integrated membrane can be better applied to guided tissue regeneration, providing more and better time and space for the regeneration of periodontal tissues and bones. This study verified the feasibility of the design through mechanical experiments, degradation experiments, electrical corrosion tests, surface morphology, and elementary composition analysis for Mg and *in vitro* cell proliferation experiments, aiming to explore the effect of the application of the Mg-reinforced PLA composite membrane in GTR/GBR.

## 2. Materials and Methods

### 2.1. Fabrication

PLA granules (Goodfellow, UK) were dissolved in a glass container containing 5% acetone, heated to 50°C, and stirred vigorously for approximately 4 h. Mg alloy AZ91 (9 wt% Al, 1 wt% Zn), both coated and bare, was applied as a reinforcement core. Coated samples were made as follows: AZ91 strips with specific size were rinsed in 35% hydrochloric acid (OCI, Korea) to clean the surface and immersed in 50% hydrofluoric acid (Duksan, Korea) for 8 h to form the fluoride coating. Then, AZ91 strips were washed using deionized water and ethanol. Finally, AZ91 strips were wholly blow-dried. Bare samples (uncoated) were treated in the same way, except for being immersed in HF (Table 1).

**Figure 1:**
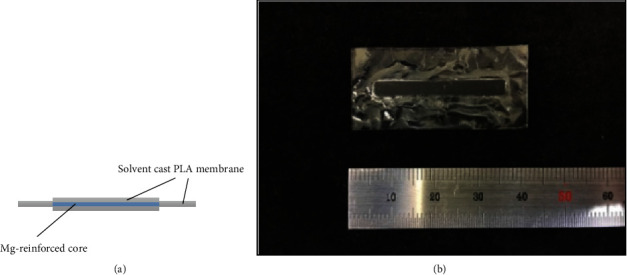
(a) Model diagram of magnesium-reinforced PLA-integrated membrane and (b) optical image of magnesium-reinforced PLA-integrated membrane after fabrication.

The PLA solution was first laid in a metal mold, followed by a Mg alloy AZ91 strip, and then a layer of PLA solution was laid on the AZ91 strip. Next, the solvent was put in a drying oven at 100°C to evaporate for 6 h and produce a Mg-reinforced PLA-integrated membrane. The fabrication method of the integrated membrane in the control group is the same as above, except that the magnesium strip was replaced by a PLA strip. The average thickness of the integrated membranes was 120 *μ*m. The integrated membranes were cut into the desired dimensions (Figure 1).

### 2.2. Mechanical Test

The dimension of the sample was 40 × 20 × 0.12 mm^3^, and the reinforcement core in the middle of the sample was 30 × 4 × 0.9 mm^3^. For experimental groups, the coated and uncoated AZ91 were used as the reinforcement cores. For the control group, the PLA strip with dimensions similar to that of the AZ91 strip was employed to replace the AZ91 as the reinforcement core.

Through the three-point bending test, the bending strength was determined as the maximum point of the load-displacement curve; this test was performed in a standard laboratory atmosphere with a universal testing machine (Instron, USA). The crosshead speed was set at 5 mm/min, and the support span was set at 20 mm. At least five samples in each group were tested.

### 2.3. Degradation Test for Membrane

The weight of magnesium strips (both coated and uncoated) used in this test was first measured as *W*_*o*_. After the fabrication procedure, coated, uncoated, and non-Mg groups were placed in the closed tubes containing 45 ml of Hank's Balanced Salt Solution (HBSS, Welgene, Korea) and incubated in water baths at 37°C. At the end of each immersion period time of 4, 8, and 12 d, five samples from coated and uncoated groups were removed from the tube. Each magnesium strip was parted from the integrated membrane. It was ultrasonically washed in 20% chromium oxide (Sigma-Aldrich, USA) solution for 1 min to clean off the oxide precipitate on the surface, rinsed with absolute ethanol (Duksan, Korea), and thoroughly blow-dried. The weight was measured again as *W*_*t*_. The weight loss percentage was calculated as follows:

(1) $$\begin{array}{c} \text{Weight loss }\left(\%\right)=\left(W_{o}–W_{t}\right)/W_{o}\times 100, \end{array}$$

where *W*_*o*_ is the initial weight, and *W*_*t*_ is the final weight.

Five samples from each group were removed from the tube and dabbed dry with the tissue, and a three-point bending test was performed on them immediately.

### 2.4. Electrochemical Corrosion Test for Mg

Coated and uncoated magnesium plates were tested by PDP and EIS tests. First, PDP test was performed on these magnesium plates; thereafter, the experimental results were analyzed using a potentiostat (VersaSTAT 3 : 300) with commercial software (VersaStudio 2.44.4). The electrochemical cell composed of a classical three-electrode cell included a particular working electrode examining sample. Pure graphite was applied as the counterelectrode and Ag/AgCl/Sat-KCl (+197 mV vs. the standard hydrogen electrode) as the reference electrode. Each magnesium plate was placed in a sealed PTFE clamp with an exposed surface area of 1 cm^2^ as the working electrode. HBSS (1000 ml) was put in a double-wall beaker as the electrolyte, and the temperature of the electrolyte was maintained at 37 ± 1°C by a circulating water heater. In this experiment, samples were immersed in the HBSS for 1 h to conduct their open circuit potential (OCP) mode. After the OCP, the PDP was done. They scanned the samples from the cathodic area to the anodic area at a rate of 1 mV/s, with a reference electrode value in the range of −2 V to −1 V. Thereafter, the corrosion potential (*E*_corr_), current density (*I*_corr_), and corrosion rate (CR) were precisely measured by the software. EIS was performed at an OCP, employing a sinusoidal potential of 5 mV in the frequency range from 10^5^ to 10^−2^ Hz.

### 2.5. Surface Morphology and Elementary Composition Analysis

The uncoated and fluoride-coated magnesium alloy AZ91 soaked in HBSS (Welgene, Korea) were taken out; and then, the magnesium alloys were separated from the integrated membranes. Scanning electron microscopy (SEM, JSM-5600; JEOL, Japan) was applied to analyze the surface microstructure and morphology of these two alloys. The concentration analysis (quantitative analysis) of the corroded surfaces and corrosion products of these two magnesium alloys was performed using EDS (Hitachi S-4800, Hitachi, Ltd., Tokyo, Japan) connected to the scanner.

### 2.6. Cell Proliferation Test

The diameter of circular specimens was 16 mm. The coated magnesium alloy-reinforced PLA-integrated membrane (4 × 4 mm) and uncoated magnesium alloy-reinforced PLA-integrated membrane were used as experimental groups. The titanium disk and PLA-integrated membrane without Mg were used as control groups. These membranes were irradiated under UV for 1 h. Then, the membrane was individually placed in one 12-well culture plate. Murine-derived preosteoblast (MC3T3-E1) and murine-derived fibroblasts (L929) cell lines were chosen. Two types of culture medium were prepared. The MC3T3-E1 cell line was cultured in a culture media consisting of Dulbecco's modified Eagle's medium (Welgene, Korea) supplemented with 10% fetal bovine serum (FBS, Gibco, USA) and 1% antibiotic/antimycotic (Gibco, USA). The L929 cell line was cultured in a culture media consisting of RPMI 1640 medium (Welgene, Korea) supplemented with 10% FBS and 1% antibiotic/antimycotic (Gibco, USA). MC3T3-E1 and L929 cells were carefully seeded on each specimen with a concentration of 1 × 10^5^ cells in 200 *μ*l of the medium and incubated in 5% CO_2_ at 37°C and 95% relative humidity for 1 h. Then, 2 ml of the medium was added to each well. Five samples in each group were cultured for 1, 3, and 5 d. The medium and plate were replaced on days 1, 3, and 5 before the WST test. At each time point, cells were assayed by WST test. 200 *μ*l of the WST assay (DoGenBio, Korea) was added to each well and incubated in a 37°C environment with 5% CO_2_ and 95% relative humidity for 3 h. Then, 100 *μ*l of the solution was transferred to a 96-well plate. The absorbance values were measured at 450 nm using the ELISA Reader. The results were expressed as the averaged absorbance levels of five replicates.

### 2.7. Statistical Analysis

Statistical analysis of the data was implemented by one-way analysis of variance (ANOVA) followed by Tukey's post hoc analysis, and a *p* < 0.05 was considered meaningful.

## 3. Results

### 3.1. Mechanical Test

**Figure 2:**
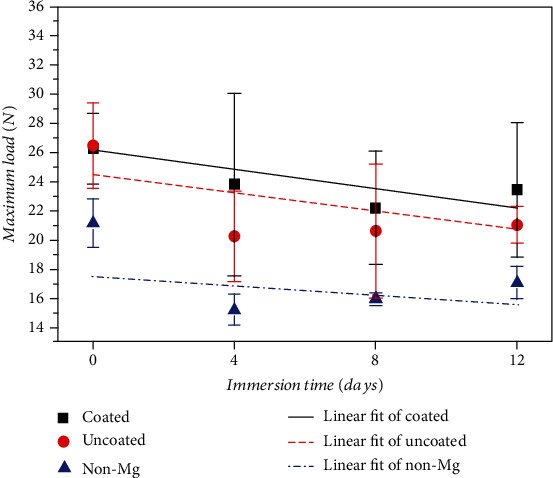
Maximum load recorded in three-point bending test after each immersion time point.

Figure 2 shows the maximum load recorded in the three-point bending test of coated, uncoated, and non-Mg groups after each immersion time point and the linear fit of each group. Maximum loads of coated and uncoated groups were higher than those of non-Mg group at all time points. Differences between coated and uncoated groups in the whole period were not significant. The trends of decreasing maximum load were similar for all groups.

### 3.2. Corrosion Tests

**Figure 3:**
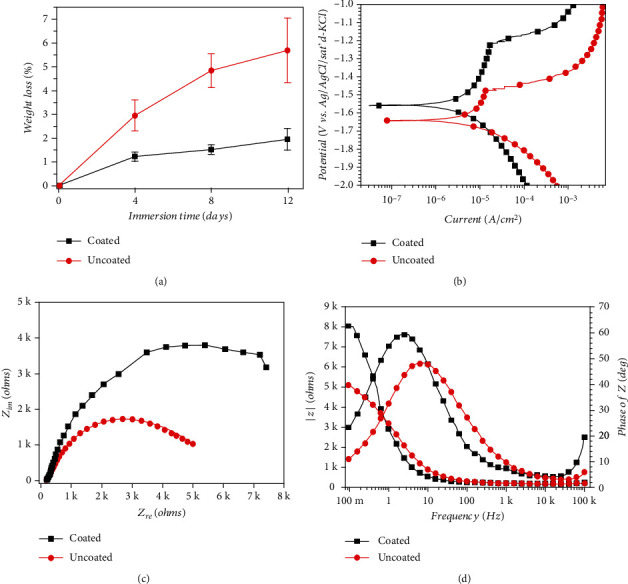
(a) Weight loss percentage of magnesium cores used in coated and uncoated groups in degradation test. (b) Potentiodynamic polarization curves for coated and uncoated magnesium plates in electrochemical corrosion test. (c) Nyquist plots of coated and uncoated magnesium plates in EIS test. (d) EIS Bode plots obtained for the coated and uncoated magnesium plates.

Figure 3(a) shows the weight loss percentage of magnesium cores used in coated and uncoated groups after immersion for 4, 8, and 12 d. The weight loss percentage of the uncoated magnesium core was high, as it reached 5.7% at day 12, while that of the coated magnesium core was much lower and was only 2.0% at day 12.  Figure 3(b) shows PDP curves for coated and uncoated magnesium plates in the PDP test. The curve of the samples showed an excursion to lower current density values when fluoride coating was applied.  Figure 3(c) shows Nyquist plots of coated and uncoated magnesium plates in EIS test. The EIS behavior of the coated magnesium alloy was distinctly different from that of the uncoated magnesium alloy. For the coated magnesium alloy, a larger capacitive loop was shown in the figure, indicating that the corrosion resistance of the coated Mg alloy was much higher than that of the untreated one.  Figure 3(d) shows EIS Bode plots obtained for the coated and uncoated magnesium plates. Under the measurement of 0.01 Hz, the resistance modulus value of the coated magnesium alloy is higher than that of the uncoated alloy.

### 3.3. Surface Morphology and Elementary Composition Analysis for Mg

**Figure 4:**
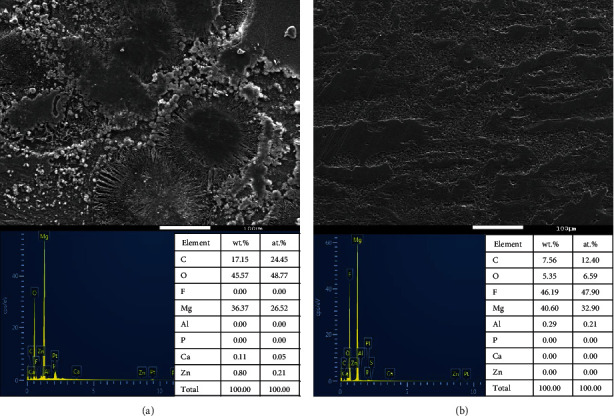
Surface morphologies (SEM) and elemental compositions (EDS) of magnesium alloys soaked for 12 days. (a) Uncoated Mg. (b) Coated Mg.

Figure 4(a) shows the corroded surface morphology and surface composition of the uncoated magnesium alloy. Through morphological scanning, it can be observed that the surface of the uncoated magnesium alloy was uneven, with prominent crevice corrosion and large-area corrosion. Moreover, a small part of the corrosion particles adheres to the surface of the alloy. From the analysis results of EDS, it can be seen that after corrosion of the uncoated magnesium alloy, from the mass fraction, O > Mg > C, and in terms of the atomic fraction, O > C > Mg.  Figure 4(b) shows the corroded surface morphology and surface composition of the coated magnesium alloy. It can be seen from the picture scanned by the SEM that the corrosion surface of the fluoride-coated magnesium alloy was flatter than that of the uncoated magnesium alloy, and a small amount of hole-type corrosion occurred. Through the EDS, the element composition of the surface of the coated magnesium alloy after 12 d of immersion can be obtained. In terms of the mass fraction, F > Mg > C > O, and as for the atomic fraction, F > Mg > C > O.

### 3.4. *In Vitro* Cell Proliferation Test

**Figure 5:**
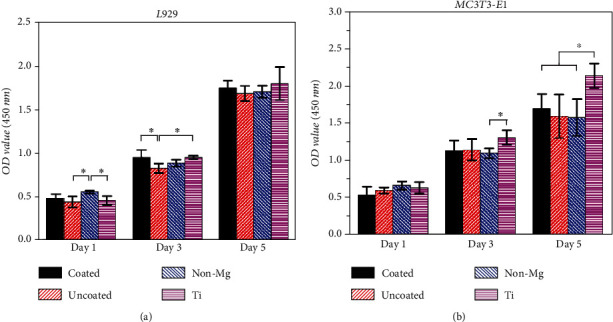
Cell proliferation on membrane materials by WST test. (a) Murine fibroblast cell L929. (b) Murine osteoblast cell MC3T3-E1. ^∗^*p* < 0.05.

Figure 5(a) shows OD value of cell proliferation test of murine fibroblast cell L929 on membrane materials by the WST test. On day 1, non-Mg group showed significantly higher OD value than those of uncoated and Ti groups. On day 3, the uncoated group exhibited fairly lower OD value than those of coated and Ti groups. On day 5, no apparent difference was shown.  Figure 5(b) shows OD value of cell proliferation test of murine osteoblast cell (MC3T3-E1) on membrane materials by the WST test. Day 1 showed no apparent difference. On day 3, the Ti group showed prominently higher OD value than that of non-Mg group. On day 5, Ti group showed relatively higher OD value than those of all other groups.

## 4. Discussions

Chen et al. [28] reported that the Young's modulus of Mg alloy was a lot closer to human bones than that of bioinert medical metals, such as Ti and stainless steel. Furthermore, the strength of Mg alloy is higher than that of bioabsorbable polymers, such as polylactic acid. Therefore, the magnesium alloy as a reinforcing core will enhance the mechanical properties of PLA and provide an excellent barrier for the regeneration of periodontal tissues. Coated groups and uncoated groups had a higher maximum load than non-Mg groups at all time points in Figure 2, indicating that the reinforcement of the magnesium core vastly increased the load capacity of the integrated membrane. Although coated groups showed close maximum loads as uncoated groups, the visual inspection after the immersion found that the corrosion was generally more severe on the magnesium cores of uncoated groups, indicating that the existence of the fluoride coating can slow down the CR of the magnesium core, and the fluoride coating is significantly necessary for this application. Since the experimental period was only 12 d, it may not be quite enough for the degradation to show the apparent influence on the strength reduction of the membranes. Thence, as shown in Figure 2, as the immersion time increased, the trends of the decrease of the maximum load were not drastic in all groups according to the linear fit. Yan et al. [29] investigated the differences between fluoride-coated and blank AZ31B after immersion in SBF. They found that both the bending strength and morphology of bare AZ31B changed even within two weeks, while fluoride-coated AZ31B had little effect. This result agreed with ours to a certain extent. Consequently, coating technique is vital for slowing down the degradation rate and mechanical property deterioration of Mg alloys when applied *in vivo*.

Metal has unique mechanical advantages, which polymer and ceramic cannot compare to. In GBR, for treating large bone defect, Ti mesh is usually required along with the support of grafting materials. The application of polymeric membranes, especially bioabsorbable ones, is considered to result in the formation of an incomplete bone structure. The degradation process of the bioabsorbable membranes may cause the inflammation and affect the healing of bone tissues, which will destroy the integrity of the reconstructed bone [30]. In the composite material of magnesium alloy and polylactic acid, when the volume fraction of magnesium alloy reached 40%, the bending strength of the composite material reached 198 MPa. However, the bending strength of pure PLA is only 88 MPa [31]. Therefore, it is undoubted that the use of AZ91 as a reinforcement core can strengthen the polymeric membrane. Besides AZ91, Mg and its other alloys also have the potential as reinforcement cores. Gu et al. [32] concluded that the fatigue strength of Mg alloys is in the range 20–100 MPa, whereas that of the polymer ranges from 15 to 58 MPa. The application of different Mg alloys may depend on their specific mechanical properties and biological safety.

The GTR/GBR membranes must not only have excellent mechanical properties but also have an appropriate degradation rate [30]. However, the degradation rate of the magnesium alloy is too fast [33]; hence, it is particularly vital to modify the surface of the magnesium alloy. Tian and Liu studied that hydrofluoric acid coating can reduce the CR of magnesium alloys, delaying the corrosion process in cell culture medium for at least one week [25]. As shown in Figure 3(a), since day 4, the degradation rate of uncoated magnesium cores was approximately two to three times more than that of the coated ones. Even with the coverage of PLA, the weight loss of the uncoated magnesium core reached approximately 6% at the end of the 12-day immersion test. Although the influence on the mechanical resistance of the membrane was not significant in this study, it can be inferred that the degradation of the magnesium core can still be a significant issue when it is implanted in the human body. Polarization testing is the most common approach to analyze corrosion performance, detecting metal corrosion potential (*E*_corr_) and the corrosion current density (*I*_corr_) in SBF. *I*_corr_ is closely related to CR. Its value is inversely correlated with corrosion resistance, while the value of *E*_corr_ is in direct proportion to the tendency of corrosion resistance. With the same potential, the coated magnesium alloy had lower current density than that of the uncoated magnesium alloy, indicating that the corrosion resistance of the magnesium alloy was enhanced by the fluoride coating in Figure 3(b). The bare Mg alloy showed weak corrosion resistance in SBF, but the corrosion was hindered after coating. The elevated corrosion resistance of coated magnesium alloy was confirmed by larger capacitive loop in Nyquist plots in Figure 3(c) and larger modulus of impedance at 0.01 Hz in EIS Bode plots in Figure 3(d). Through the comparative observation of the surface morphology shown in Figures 4(a) and 4(b), it can be found that the existence of the fluoride coating has hindered the CR of the magnesium alloy and significantly reduced the corrosion efficiency of the magnesium alloy. This experimental result proved that the fluoride coating played a crucial role in decreasing the CR of the magnesium alloy, and this result was consistent with that of previous studies [34, 35]. The energy spectrum analysis charts in Figures 4(a) and 4(b) showed that the corrosion products of magnesium alloys soaked for 12 d were mainly oxygen-containing compounds. It can be seen that the deposition of corrosion products of the coated magnesium alloy was significantly less than that of the uncoated magnesium alloy. On the surface of the uncoated magnesium alloy, the reaction acted as follows:

(2) $$\begin{array}{c} \text{Mg}+{\text{H}}_{2}\text{O}⟶\text{Mg}{\left(\text{OH}\right)}_{2} \end{array}$$

(3) $$\begin{array}{c} \text{Mg}{\left(\text{OH}\right)}_{2}⟶\text{MgO}+{\text{H}}_{2}\text{O} \end{array}$$

The surface of the coated magnesium alloy was mainly composed of MgF_2_, which is not easily soluble in water due to the presence of the fluorine. Consequently, there were few corrosion products on the surface of the coated magnesium alloy AZ91, and the degree of the corrosion was lighter. These experimental results in Figures 3 and 4 demonstrated the importance of fluoride coatings for magnesium alloys as implants *in vivo*.

The superior biocompatibility was one of the vital characteristics of implants *in vivo* [36]. As shown in Figure 5(a), fibroblast cells retained steady growth on the coated groups, which can be compared to that on the Ti groups. Although there was some fluctuation in non-Mg and uncoated groups on days 1 and 3, all groups showed similar cell proliferation on day 5. The cell affinity of fibroblast cells to membrane materials can infer the materials' biocompatibility when membranes were implanted into the human body. Furthermore, the cytocompatibility of PLA and magnesium had excellent cell compatibility. In Figure 5(b), osteoblast cells grew better on the titanium, especially on day 5. As is well known, titanium has the feature of osseointegration, which can well explain its better affinity to osteoblast cells. Except for the Ti groups, osteoblast cells had similar proliferation rate on all PLA-integrated membranes with or without the reinforcement of magnesium. These results indicate that the addition of the magnesium core does not harm osteoblast cells in the early stage. Although it cannot be compared to titanium, the cell affinity of PLA has been proven to be sufficient for medical applications and as the GBR membrane. Therefore, this magnesium-reinforced PLA-integrated membrane has passed the trial on both fibroblast and osteoblast. It is going to need an *in vivo* test to confirm the integrated membrane in the future [37].

## 5. Conclusion

In this study, a GBR/GTR-integrated membrane made of PLA and reinforced by an Mg alloy core was fabricated. The integrated membrane had better load capacity compared to those without the Mg reinforcement. When fluoride-coated magnesium alloy was used, it showed an appropriate degradation rate and better corrosion resistance. Osteoblast and fibroblast cells both grew well on it. Consequently, this bioresorbable magnesium-reinforced PLA-integrated membrane has a potential application in the GBR/GTR technique.

## Figures and Tables

**Table 1:** Group codes and referred composition of samples.

Group	Composition
Coated	Fluoride-coated AZ91-reinforced PLA-integrated membrane
Uncoated	Uncoated AZ91-reinforced PLA membrane
Non-Mg	Integrated membrane with PLA strip
Ti	Titanium disk

## Data Availability

The data used to support the findings of this study are included within the article.
